# Radiation-Guided Peptide Delivery in a Mouse Model of Nasopharyngeal Carcinoma

**DOI:** 10.1155/2016/5382047

**Published:** 2016-09-21

**Authors:** Pei-cheng Lin, Jun-yan He, Yu-yin Le, Kai-xin Du, Wei-feng Zhu, Qing-qin Peng, Ya-ping Dong, Jin-luan Li, Jun-xin Wu

**Affiliations:** ^1^Department of Radiation Oncology, Teaching Hospital of Fujian Medical University, Fujian Provincial Cancer Hospital, Fuzhou, China; ^2^Fuzhou Pulmonary Hospital of Fujian, Fuzhou, China; ^3^Department of Pathology, Teaching Hospital of Fujian Medical University, Fujian Provincial Cancer Hospital, Fuzhou, China

## Abstract

*Purpose.* This study aimed to evaluate the characteristics of the HVGGSSV peptide, exploring radiation-guided delivery in a mouse model of nasopharyngeal carcinoma.* Methods.* Mice with CNE-1 nasopharyngeal carcinoma were assigned to two different groups treated with Cy7-NHS and Cy7-HVGGSSV, respectively. Meanwhile, each mouse received a single dose of 3 Gy radiation. Biological distribution of the recombinant peptide was assessed on an* in vivo* small animal imaging system.* Results.* The experimental group showed maximum fluorescence intensity in irradiated tumors treated with Cy7-labeled HVGGSSV, while untreated (0 Gy) control tumors showed lower intensity levels. Fluorescence intensities of tumors in the right hind limbs of experimental animals were 7.84 × 10^7^ ± 1.13 × 10^7^, 1.35 × 10^8^ ± 2.66 × 10^7^, 4.05 × 10^8^ ± 1.75 × 10^7^, 5.57 × 10^8^ ± 3.47 × 10^7^, and 9.26 × 10^7^ ± 1.73 × 10^7^ photons/s/cm^2^ higher compared with left hind limb values at 1, 2, 15, 24, and 48 h, respectively. Fluorescence intensities of tumor in the right hind limbs of the experimental group were 1.66 × 10^8^ ± 1.71 × 10^7^, 1.51 × 10^8^ ± 3.23 × 10^7^, 5.38 × 10^8^ ± 1.96 × 10^7^, 5.89 × 10^8^ ± 3.57 × 10^7^, and 1.62 × 10^8^ ± 1.69 × 10^7^ photons/s/cm^2^ higher compared with control group values at 1, 2, 15, 24, and 48 h, respectively. Fluorescence was not specifically distributed in the control group. Compared with low fluorescence intensity in the heart, lungs, and tumors, high fluorescence distribution was found in the liver and kidney at 48 h.* Conclusions.* HVGGSSV was selectively bound to irradiated nasopharyngeal carcinoma, acting as a targeting transport carrier for radiation-guided drugs that are mainly metabolized in the kidney and liver.

## 1. Introduction

Although concurrent chemoradiotherapy has become an important treatment modality for cancer [1], nonspecific targeting damage of normal cells and organs increases the overall toxicity [2]. Therefore, developing a targeted radiation carrier that guides chemical drugs to the tumor has attracted significant attention [3–5].

Peptides are widely used in targeted drug carrier research due to high affinity and specificity, low molecular weight, lack of immunogenicity, and absorbability by the tumor cells [6–8]. Lowery et al. [9] and Passarella et al. [10] identified recombinant peptides (GIRLRG, AARLY, HMWRDSQ, and HVGGSSV) that could specifically bind irradiated tumors using the phage display technology. Wang et al. [11] revealed that HVGGSSV could specifically bind to tumor cells injured by radiation. Furthermore, increased expression of the PDZ-TIP-1 protein, which is a specific recognition ligand of HVGGSSV, might be the underlying mechanism.

As proposed by Hariri et al. [12], we synthesized the recombinant peptide HVGGSSV, which was then labeled with Cy7-N-hydroxysuccinimide (Cy7-NHS) ester. Our preliminary results demonstrated that red fluorescence signals were localized and persisted within the irradiated region of the tumor, whereas low intensity fluorescence was observed in unirradiated tumors and normal organs. These findings suggested that HVGGSSV might selectively bind to irradiated tumors. Herein, we aimed to further validate the specific targeting ability of this polypeptide, which might provide an experimental basis for subsequent research of drug-targeting carriers. Our data might also promote novel designs for evaluating radiotherapy efficacy and imaging diagnosis.

## 2. Material and Methods

### 2.1. Cell Culture and Tumor Model

Human nasopharyngeal carcinoma CNE-1 cells were provided by Tumor Radiation Biological Laboratory of Fujian Provincial Tumor Hospital and cultured in complete RPMI 1640 at 37°C and 5% CO_2_, in a humid environment. Five-week-old male nude mice weighing 18–20 g were anesthetized by intraperitoneal injection of chloral hydrate. A 0.1 mL cell suspension (1 × 10^7^/mL) was inoculated subcutaneously in bilateral hind limbs of each animal. Once the tumor had reached approximately 1 cm in diameter (after 1-2 weeks), the mice were subjected to* in vivo* experiments.

### 2.2. Cy7 Mono NHS Ester Labeling of HVGGSSV

The fluorescent molecule Cy7 mono NHS ester was conjugated to the peptide to yield the Cy7-HVGGSSV fluorescence polypeptide, which was purified by SephadexG-15. After buffer elution, two bands were identified: the first outflow was the labeled conjugate Cy7-HVGGSSV while the band atop the column was free Cy7. The samples were freeze-dried for 66 h, and absorption spectra of the Cy7-HVGGSSV compound were measured on a spectrophotometer.

### 2.3. *In Vivo* Carrier Targeting Distribution

Mice were randomly divided into two groups (*n* = 8) and received 3 Gy radiation to the right hind limb tumor. 4 h after irradiation, control group mice were administered with 0.1 mL of free Cy7 solution (1 mg/L) to tail vein while the mice in the experimental group were injected with 0.1 mL of Cy7-HVGGSSV solution (1 mg/L) similarly.

Mice were anesthetized and fastened in a thermoplastic membrane. Right hind limb tumors and outer margins (0.5 cm around) were considered the radiation field, while left hind limb tumor was avoided. The mice were subjected to 3 Gy dose on a 6 MV X-ray linear accelerator.

### 2.4. Fluorescence Carrier Targeting Combined with Radiation-Injured Tumors

Fluorescence distribution in bilateral hind limb tumors was assessed on a small animal imaging system* in vivo*. The mice were fasted for 6 h to reduce interference of the gastrointestinal content. Subsequently, the animals were anesthetized by chloral hydrate and subjected to imaging after drug injections at 1, 2, 15, 24, and 48 h, respectively. Maximum excitation wavelength was 747 nm, for emission at 775 nm. Bilateral limb tumors were selected in fluorescent images as the region of interest (ROI). Then, fluorescence quantum in the ROI was measured, and fluorescence intensity of the tumor area was analyzed and compared in bilateral limbs. Changes in fluorescence quantum at different time points in the same ROI were monitored.

### 2.5. Fluorescence Carrier Distribution and Metabolism in the Organs

Because of the complicated mouse structure and composition, hair, necrotic tumor tissue, and food in the gastrointestinal tract may produce spontaneous fluorescence that increases the background noise, affecting the recognition of specific fluorescent substances. To reduce such interference, the mice were sacrificed at 48 h after drug administration, and hearts, livers, lungs, kidneys, and tumors were excised and assessed on a small animal imaging system* in vivo*.

### 2.6. Small Animal Imaging System* In Vivo*

Pseudocolor and scanning images for each mouse were overlaid and fused by the small animal imaging system* in vivo* [20, 21]. The fusion image color reflected the* in vivo* distribution of the fluorescence polypeptide. Orange indicated the strongest fluorescence intensity at 750 nm excitation, with highest content of the fluorescent molecule Cy7, whereas purple reflected a very low fluorescence intensity. The color spectrum of orange, red, yellow, green, blue, and purple indicated high to low fluorescence intensity in that order.

### 2.7. Data Analysis and Statistics

Data are mean ± standard deviation (SD) and were analyzed by the SPSS 19.0 software. Differences between groups were assessed by *t*-test. *P* < 0.05 was considered statistically significant.

## 3. Results

### 3.1. Fluorescence Spectrum Analysis

**Figure 1:**
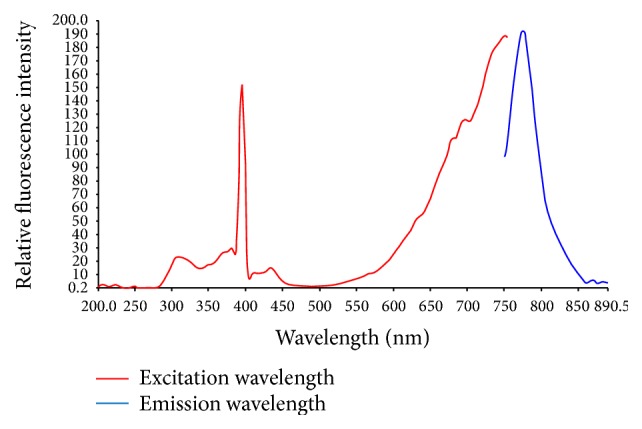
Fluorescence spectrum analysis. The red line shows the excitation wavelength of Cy7-HVGGSSV; the blue line is emission wavelength.

Maximum excitation wavelength was 747 nm, for a maximum emission wavelength of 775 nm. Spectroscopic results are shown in Figure 1. Excitation and emission wavelengths modestly varied from those of free Cy7 [13, 14] as previously described; this was caused by a slight shift of emission and excitation peaks after labeling with the fluorescent probe.

### 3.2. *In Vivo* Fluorescent Polypeptide Targeting Combined with Tumor Radiation Injury

**Figure 2:**
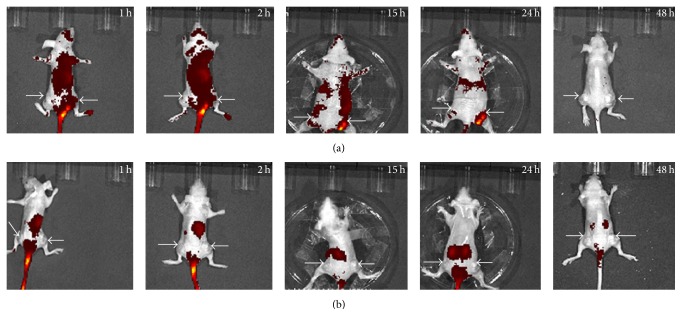
*In vivo* fluorescence distribution images. (a) shows the fluorescence of Cy7-HVGGSSV. At 15–24 hours after treatment, orange fluorescent signals began to accumulate in right hind limb tumors. No obvious orange fluorescence in left hind limb tumors was found. (b) shows the fluorescence of the control group.

Cy7-HVGGSSV and free Cy7 fluorescent molecules (control) were administered by tail vein injection. Then, distribution and metabolism of the polypeptide were assessed on an* in vivo* small animal imaging system (Figure 2).

Figure 2(a) shows Cy7-HVGGSSV fluorescence. At 1-2 h after polypeptide injection, Cy7-HVGGSSV was evenly distributed throughout the body, and no orange signal was apparent in tumors of bilateral hind limbs. At 15–24 h after treatment, the orange fluorescence began to accumulate in right hind limb tumors, whereas such signal was absent in left hind limbs.

**Figure 3:**
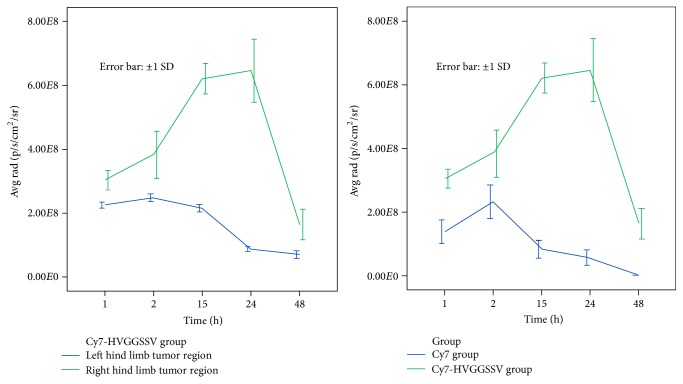
Differences in fluorescence signals in various sites.

According to the linear distribution theory of fluorescence intensity, fluorescence intensities in other body areas were significantly different from values in right hind limb tumors, which were remarkably lower compared with those in tumors treated by radiation injury. Fluorescence signal data in tumors of the Cy7-HVGGSSV group are shown in Table 1. Variations in fluorescent signals between the bilateral hind limbs are depicted in Figure 3. At 15–24 h, mean fluorescent signal distributions in right hind limb tumors were 4.05 × 10^8^ ± 1.75 × 10^7^ and 5.57 × 10^8^ ± 3.47 × 10^7^ photons/s/cm^2^ higher compared with left hind limb values. No fluorescence distribution was found in experimental mice after 48 h. Figures 2 and 3 show the dynamic changes of fluorescent signals from strong to weak in all groups; fluorescence signals in the Cy7-HVGGSSV group peaked between 15 and 24 h.

Cy7 group results are shown in Figure 2(b). No overt orange signal was obtained in any body part of Cy7 group animals, and fluorescence distribution showed no specificity or intensity differences among bilateral hind limb tumors. The tumor area in the right hind limb was sketched as the ROI, and fluorescence signal was measured (Table 2). Figure 3 shows that the free fluorescent molecule Cy7 was evenly distributed throughout the body at 1-2 h after injection. Pronounced red signals were not seen in the tumor sites of bilateral hind limbs. Since the fluorescent molecule was quickly removed from the blood, its distribution in mice was reduced. In images scanned at 15, 24, and 48 h, fluorescence intensities gradually decreased, and no high signal was observed in the tumor areas of bilateral hind limbs (*P* ≥ 0.05). Mean fluorescence intensities of right hind limb tumors of the Cy7 group were 1.66 × 10^8^ ± 1.71 × 10^7^, 1.51 × 10^8^ ± 3.23 × 10^7^, 5.38 × 10^8^ ± 1.96 × 10^7^, 5.89 × 10^8^ ± 3.57 × 10^7^, and 1.62 × 10^8^ ± 1.69 × 10^7^ photons/s/cm^2^ lower compared with Cy7-HVGGSSV group values at 1, 2, 15, 24, and 48 h, respectively.

### 3.3. Fluorescence Carrier Distribution throughout the Body

**Figure 4:**
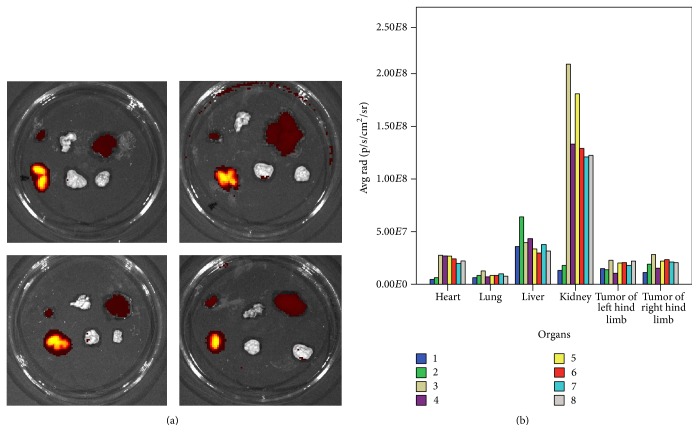
Fluorescence distribution of Cy7-HVGGSSV* in vivo*. (a) Fluorescence images of separated organ in the Cy7-HVGGSSV group are shown (top row from left to right are heart, lungs, and liver; bottom row from left to right are kidneys, left hind limb tumor, and right hind limb tumor). Kidneys showed the strongest fluorescence intensity, followed by liver. (b) Each number represents an experimental group mouse. Fluorescence signals of mice (from left to right: hearts, lungs, livers, kidneys, and tumors in left and right hind limbs) in the Cy7-HVGGSSV group are shown. Fluorescence carrier intensity from strongest to weakest was kidney > liver > right hind limb tumor > left hind limb tumor > lung > heart.

Mice were sacrificed at 48 h after drug administration, and hearts, livers, lungs, kidneys, and tumors were excised for assessment on a small animal imaging system* in vivo*. Fluorescence signals and fluorescence image of separated organ in mice of the Cy7-HVGGSSV group are shown in Figure 4. Fluorescence intensities from strongest to weakest were kidney > liver > tumor in right hind limb > tumor in left hind limb > lung > heart. Cy7-HVGGSSV was distributed mainly in the kidney and liver, with low fluorescence signals in the other organs as well as bilateral hind limb tumors.

## 4. Discussion

This study assessed the biological features and drug loading properties of HVGGSSV, a new nanometer-sized targeting carrier. Using a small animal imaging system, distribution and metabolism of Cy7-labeled HVGGSSV were observed* in vivo* and its binding characteristics to tumors after radiation injury were evaluated. On fluorescence images of tumor-bearing nude mice treated with Cy7-labeled HVGGSSV, fluorescent peptides were accumulated in irradiated right hind limb tumors, while less fluorescence was observed in nonirradiated left hind tumors. Substantially higher fluorescence signals were obtained in the liver and kidney compared with heart and lung values 48 hours after injection, suggesting that the polypeptide might be degraded in the liver and kidney, thereby avoiding side effects on other tissues. According to the instructions of small animal imaging system* in vivo*, fluorescence intensities of different regions could only be compared in the same background. To compare fluorescence signals at different times, both fluorescence intensity and quantum should be taken into consideration. Additionally, to highlight the differences among various regions, with this peptide mainly metabolized in the kidney, different orders of magnitude were, respectively, used in Figures 2 and 4 (10^9^ versus 10^8^). Our results showed that the targeting carrier could accumulate in irradiated tumors and might be metabolized in the kidney and liver.

The phage display technology described here allowed us to examine polypeptides that specifically bind to tumors. It could rapidly screen bioactive peptides, proteins, receptors, and other novel or lead compounds with high stability and specificity in the body [15, 16]. However, the phage display technology has a limited ability to detect* in vivo* and* in vitro* differences in polypeptide metabolism or differences in affinity and clearance between polypeptides and targeted proteins. The small animal imaging system can directly assess the labeled experimental subjects in real time and* in situ*, rapidly yielding quantitative information in a completely* in vivo* environment.

The molecular weights of polypeptides are lower than antibody values (1-2 kDa). Therefore, polypeptides have better bioavailability and permeability, with higher safety in the tumor tissue. Moreover, they also exhibit accelerated metabolism in the body. Their limitations include instability on the tumor cell surface and alterations according to the environment [17, 18]. Sugahara et al. found a handful of peptides that could specifically interact with the tumor cell surface, constituting ideal carriers of targeted therapy [19]. Lowery et al. identified the HVGGSSV peptide using the phage display technology; it could specifically target lung cancer cells with radiation. Consequently, HVGGSSV is considered a chemotherapeutic drug-targeting carrier that could combine the curative effect of cytotoxic drugs with tumor targeting and be used as a carrier to maximize patients' benefit from chemotherapy [9]. Wang et al. [11] revealed that HVGGSSV could specifically bind to radiation-injured tumor cells, the primary principle of which is that radiation could increase PDZ-TIP-1 expression on tumor cell and vessel surfaces. PDZ-TIP-1 is a specific recognition ligand of HVGGSSV, enabling it to recognize and bind to radiation-injured tumor tissues.

Herein, we synthesized HVGGSSV according to Lowery et al. [9], with the CGGGKKKGGGNHVGGSSV sequence. This polypeptide was cross-linked through a disulfide bond between two cysteines, and the ring bound the target protein. Our results confirmed that HVGGSSV had the potential to interact specifically with radiation-injured tumors, distributing to a very low degree in the surrounding tissues.

Fluorescence distribution patterns in bilateral hind limb tumors between the Cy7-HVGGSSV and Cy7 groups at 1 or 2 h were similar. At 15 and 24 h after treatment, red signals were clearly visible in right hind limb tumors in Cy7-HVGGSSV group mice, while no signal was visualized in the corresponding right hind limb tumors of Cy7 group animals. These results suggested that the distribution of free fluorescent Cy7 molecule is not restricted. Fluorescent signals in the left hind limb tumor area were lower and dissipated rapidly compared to those in right hind limbs in Cy7-HVGGSSV group animals, suggesting that distribution of the HVGGSSV polypeptide was higher in radiation-damaged tumors.

No signals were observed in Cy7-HVGGSSV or Cy7 group mice at 48 h. In addition, Lowery et al. [9] found fluorescence in the radiation-injured tumor area at 72 h after treatment. This may be attributable to HVGGSSV usage in building a paclitaxel liposome targeting carrier, the molecular weight of which was higher, and the liposome could delay particle removal. Hence, no fluorescence distribution was observed in this study at 48 h. The following reasons might explain these findings: (1) the fluorescent polypeptides and free fluorescent molecules gradually diminished with catabolism in the blood; (2) the fluorescent dye was distributed in deep viscera of the body, and emission wavelength could not penetrate the animals, making it necessary to observe the signal distribution in murine organs.

Unlike the current study, Lowery et al. [9] did not explore the distribution of fluorescent polypeptides in body organs. Although fluorescence was directly observed on the body surface after nasopharyngeal cancer treatment with the recombinant polypeptide HVGGSSV, distribution of the fluorescent polypeptides in mouse organs* in vivo* could not be quantitatively evaluated due to interference by objective factors. Mice were sacrificed after 48 h of* in vivo* observation; the organs were then excised to assess polypeptide distribution. Our results showed the following order for polypeptide distribution: kidney > liver > right hind limb tumor > left hind limb tumor > lung > heart. Fluorescence distribution in the kidney was markedly persistent, suggesting that this carrier was primarily metabolized in the kidney, largely damaging this organ. Fluorescence distribution in the liver was lower than that in the kidney; meanwhile, right hind limb tumor signals were higher than left limb values. These findings suggested that the fluorescence carrier persisted in right hind limb tumors. Since the fluorescence carrier was cleared from the body, it could not be definitely claimed that HVGGSSV was targeted to radiation-injured tumors. The current data showed that this peptide might be metabolized in kidney, followed by liver; therefore, hepatotoxicity and nephrotoxicity constitute the focus of future research in our laboratory.

The current study reported the successful building of HVGGSSV, a targeting carrier with adequate biological compatibility and high binding capacity with irradiated nasopharyngeal carcinoma. HVGGSSV is anticipated to become an efficient transport carrier of targeted therapeutics for cancer, thus contributing towards an advanced approach for an ideal combination model of chemotherapy and radiotherapy.

## Figures and Tables

**Table 1:** Fluorescence signaling results in Cy7-HVGGSSV group tumors.

Time	Group	Mean value (photons/sec/cm^2^)	Standard deviation
1 H	Left hind limb tumor region	2.26 × 10^8^	9.30 × 10^8^
	Right hind limb tumor region	3.04 × 10^8^	3.05 × 10^7^

2 H	Left hind limb tumor region	2.48 × 10^8^	1.19 × 10^7^
	Right hind limb tumor region	3.83 × 10^8^	7.42 × 10^7^

15 H	Left hind limb tumor region	2.15 × 10^8^	1.22 × 10^7^
	Right hind limb tumor region	6.20 × 10^8^	4.79 × 10^7^

24 H	Left hind limb tumor region	8.79 × 10^7^	7.63 × 10^6^
	Right hind limb tumor region	6.45 × 10^8^	9.80 × 10^7^

48 H	Left hind limb tumor region	7.07 × 10^7^	1.03 × 10^7^
	Right hind limb tumor region	1.63 × 10^8^	4.79 × 10^7^

**Table 2:** Time comparison of fluorescence signals of right hind limb tumors.

Time	Group	Mean value (photons/sec/cm^2^)	Standard deviation
1 H	Cy7 group	1.38 × 10^8^	3.76 × 10^7^
	Cy7-HVGGSSV group	3.04 × 10^8^	3.05 × 10^7^

2 H	Cy7 group	2.32 × 10^8^	5.34 × 10^7^
	Cy7-HVGGSSV group	3.83 × 10^8^	7.42 × 10^7^

15 H	Cy7 group	8.28 × 10^7^	2.78 × 10^7^
	Cy7-HVGGSSV group	6.20 × 10^8^	4.79 × 10^7^

24 H	Cy7 group	5.61 × 10^7^	2.39 × 10^7^
	Cy7-HVGGSSV group	6.45 × 10^8^	9.80 × 10^8^

48 H	Cy7 group	9.02 × 10^7^	1.69 × 10^7^
	Cy7-HVGGSSV group	1.63 × 10^8^	4.79 × 10^7^
